# Supplementary material for: Long-term experimental evolution of HIV-1 reveals effects of environment and mutational history
Source: PLoS Biol. 2020 Dec 28;18(12):e3001010. doi: 10.1371/journal.pbio.3001010 (PMC7793244; doi:10.1371/journal.pbio.3001010)
Supplement: S1 Text — (PDF) [file pbio.3001010.s001.pdf]

Supplemental Material to "Long-term experimental evolution of HIV-1 reveals effects of environment and mutational history"

1 Contamination in experiment I of the HIV Long Term Evolution Experiment.

1.1 Deletions in the Nef-gene

Throughout all but one of the experiments, we find large deletions in the nef-gene. This is not surprising, since nef is not needed for replication in cell culture (Kestler et al., 1991). While deletions are common in all experimental lines, a striking pattern occurs between the same-environment lines of the first experiment (MT-2.A/MT-2.B and MT-4.A/MT-4.B) and their derived lines. The exact same deletions (same start- and end positions) are detected in lines that are supposed to be evolutionary independent (Figure A)

In both cases, there is a very large (200bp+) deletion that is shared between the same-environment lines. Some smaller deletions are also shared. We consider it exceedingly unlikely that these deletions occurred independently, and must thus assume that a contamination happened between MT-2.A/MT-2.B and MT-4.A/MT-4.B. Considering the experimental setup, this can indeed not be excluded. The corresponding cultures were located next to each other on the plates, and simultaneously open during a transfer. Contamination between the different environments is less likely, since these parts of the plate were not simultaneously open for transfer. In later experiments, individual flasks were used to prevent contamination.

1.2 Consequences for data-analysis

Mutations occuring in the affected evolutionary lines can not be considered independent events, since they might have arrisen only once and spread to the other line via the contamination.

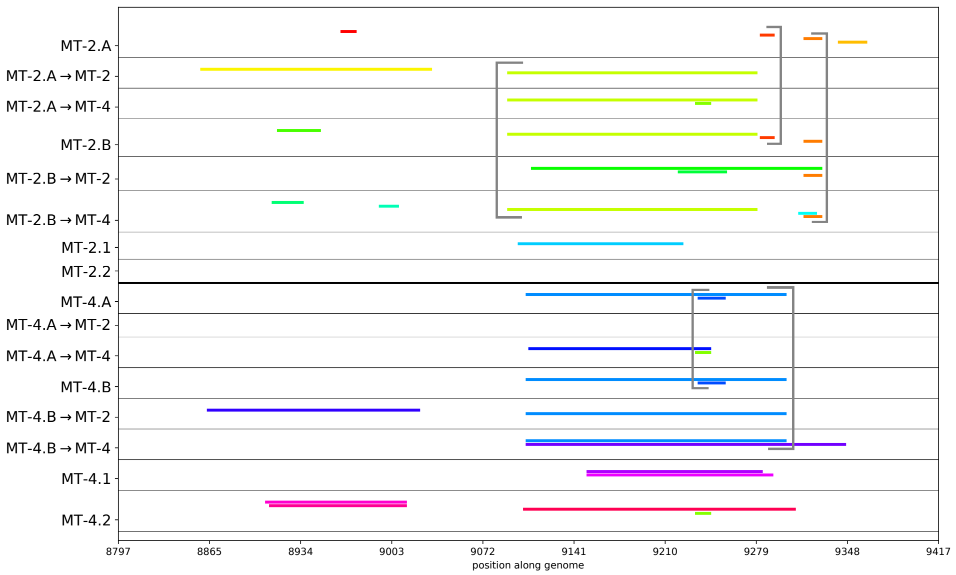

Figure A: Location of large deletions in the nef gene of all experimental lines. Colored lines indicate deleted segments at some time during the experiment. Lines with the same color have the same start- and end position. Grey brackets indicate deletions that are shared, likely due to a contamination.

1.2.1 Shared & unique mutations

Mutations were only considered shared if they independently occured in two or more evolutionary lines. They are only considered unique if they occur in a single line only.
More completely:

1. Mutations occuring both (and only) in MT-2.A/MT-2.B or MT-4.A/MT-4.B are not considered independent or shared. Only if they occur in another, independent line do they count towards the shared statistic.

2. Mutations occuring in the derived lines are only considered independent from their parental (or contamination partner) lines if they first occurred after these lines were initiated (the mutation must be absent or within the range of sequencing error – at a frequency below 1% – at passage 50 of the parental line)

1.2.2 Always and never combinations

In order for an always-combination to count, it must occur between two independent lines. We therefore only used replicates 1,3 and 4 of both environments for this measure. For never combinations, relatedness is not important – since new mutations constantly appear,

a combination of two mutations that are not observed together in multiple lines is a signal for negative epistasis, regardless of shared history.

1.3 Methods: detecting large deletions

Mapping software is reasonably good at detecting small deletions (<5bp), but larger deletions are difficult to accurately identify. Large deletions were therefore identified via a manually guided algorithm based on the coverage in the nef-gene. Coverage graphs were plotted per evolutionary line (see figure B) and gaps (sudden drops in coverage) were manually determined by zooming in and finding the exact position where the coverage drops. The program then looked for drops in coverage in the same region in other evolutionary lines. These hits were then manually checked if they truely share the same start and end position.

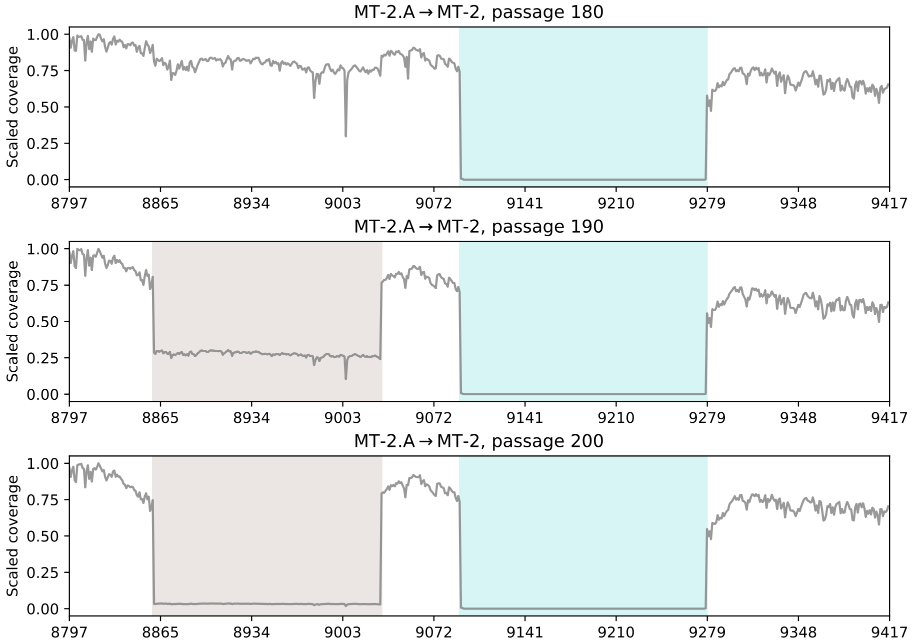

Figure B: Graph of scaled coverage (number of reads divided by the maximum coverage across the nef-gene) for 3 sequence samples in line MT-2.A→MT-2. Shaded areas indicate deletions automatically identified as present, after the start- and end positions were manually determined.

References

Kestler H.W., Ringler D.J., Mori K., Panicali D.L., Sehgal P.K., et al. Importance of the nef gene for maintenance of high virus loads and for development of AIDS. Cell, 65(4):651–662, 1991.
