# Supplementary material for: Long-term experimental evolution of HIV-1 reveals effects of environment and mutational history
Source: PLoS Biol. 2020 Dec 28;18(12):e3001010. doi: 10.1371/journal.pbio.3001010 (PMC7793244; doi:10.1371/journal.pbio.3001010)
Supplement: S2 Text — (PDF) [file pbio.3001010.s002.pdf]

Supplemental Material to "Long-term experimental evolution of HIV-1 reveals effects of environment and mutational history"

2 Effects of APOBEC and ZAP in the HIV Long Term Evolution Experiment.

Proteins from the APOBEC family are parts of the innate immune system, causing hypermutation in single-stranded DNA, typically inactivating the virus by creating lethal mutations. Especially APOBEC3G, a deaminase causing G→A mutations, is often cited as causing extensive hypermutation in HIV genomes. HIV, however, has its own protection mechanism against APOBEC in the form of the vif protein, which is capable of completely deactivating APOBEC activity.

Of the two cell lines used in our experiment, only MT-2 expresses APOBEC3G. However, the HIV strain used has a fully competent *vif* gene, leading to no loss in growth rate due to APOBEC (see Figure A), while there is a significant loss in vif-deficient virus growing on MT-2, but not MT-4.

Since there is evidence of APOBEC3G being able to occasionally cause non-lethal hypermutation in the presence of vif (Sadler et al., 2010), we also compared the mutation frequencies between MT-2, where APOBEC3G is present, and MT-4, where it is not. Should there be a 'spillover' effect of APOBEC3G then we should see an overrepresentation of G→A mutations in MT-2, especially within the APOBEC3G context. However, we do not see a difference in frequency of these mutations throughout the experiment (Figure B).

Another innate immune protein is ZAP (zinc-finger antiviral protein), which binds to CpG sites in the genome in order to inhibit replication (Ficarelli et al., 2020). In order to avoid the effect of this protein, the HIV genome is already greatly depleted in CpG sites (Alinejad-Rokny et al., 2016). We see no significant change in CpG frequency at the final time point compared to the ancestor (see Figure C). In fact, while almost all other

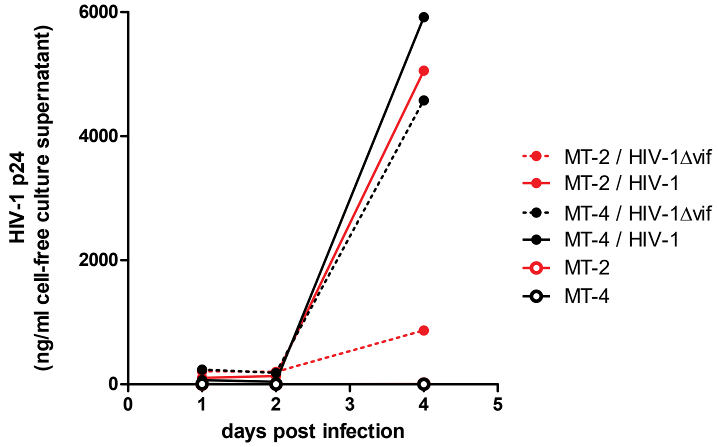

Figure A: Replication kinetics of HIV-1 NL4-3 (solid lines) and HIV-1 NL4-3 Δvif (dotted lines) in MT-2 (red lines and symbols) and MT-4 (black lines and symbols) cells. Cells were infected with an MOI of 0.01. Cell-free supernatants were collected at days 1, 2, and 4 post infection and viral p24 antigen was quantified by enzyme-linked immunosorbent assay.

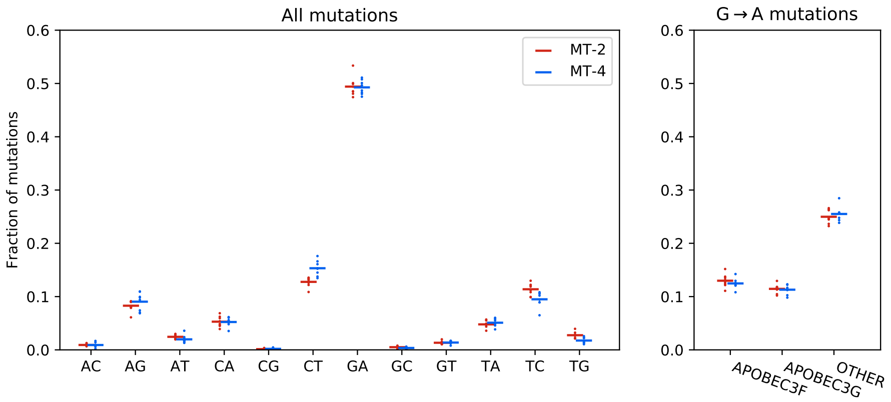

Figure B: Frequency of different mutations in the dataset. Left: breakdown of all mutations by mutation type. Right: breakdown of G→A mutations by sequence context (APOBEC3F context: **G**GD, APOBEC3G context: **G**AD, where D is any of A,T or G (Beale et al., 2004)). Every dot represents the fraction of mutations that reach a frequency of 0.05 or more of this type in each line, the lines indicate the mean within this group. None of the mutation types have significantly different mutation fractions between MT-2 and MT-4

dinucleotide frequencies change in line with the nucleotide frequencies (which change due to somewhat uneven mutation rates), CpG remains remarkably constant, indicating that those remaining CpG sites are likely conserved sites.

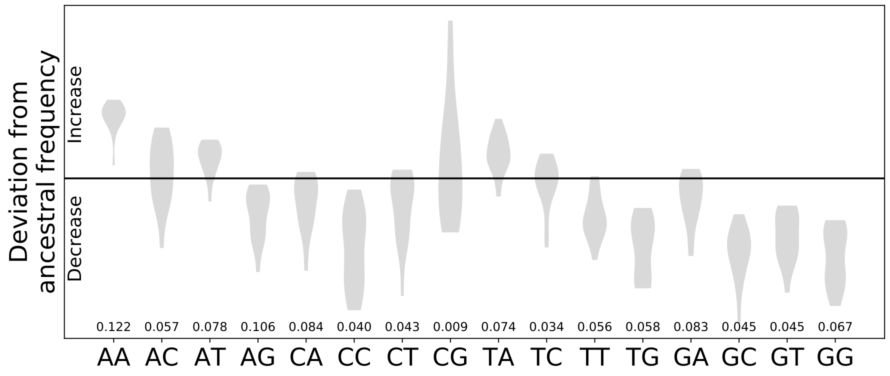

Figure C: Change in dinucleotide frequencies from the ancestor to passage 240. Violinplots indicate the spread of change (final frequency devided by initial frequency) for all the evolutionary lines. Horizontal line is at 1 (no change). The numbers below show the ancestral frequency of this dinucleotide.

References

Alinejad-Rokny H., Anwar F., Waters S.A., Davenport M.P., Ebrahimi D. Source of CpG Depletion in the HIV-1 Genome. Mol. Biol. Evol., 33(12):3205–3212, 2016.

Beale R.C., Petersen-Mahrt S.K., Watt I.N., Harris R.S., Rada C., et al. Comparison of the Differential Context-dependence of DNA Deamination by APOBEC Enzymes: Correlation with Mutation Spectra in Vivo. J. Mol. Biol., 337(3):585–596, 2004.

Ficarelli M., Antzin-Anduetza I., Hugh-White R., Firth A.E., Sertkaya H., et al. CpG Dinucleotides Inhibit HIV-1 Replication through Zinc Finger Antiviral Protein (ZAP)-Dependent and -Independent Mechanisms. J. Virol., 94(6), 2020.

Kestler H.W., Ringler D.J., Mori K., Panicali D.L., Sehgal P.K., et al. Importance of the nef gene for maintenance of high virus loads and for development of AIDS. Cell, 65(4):651–662, 1991.

Sadler H.A., Stenglein M.D., Harris R.S., Mansky L.M. APOBEC3G Contributes to HIV-1 Variation through Sublethal Mutagenesis. J. Virol., 84(14):7396–7404, 2010.
