# Supplementary material for: Long-term experimental evolution of HIV-1 reveals effects of environment and mutational history
Source: PLoS Biol. 2020 Dec 28;18(12):e3001010. doi: 10.1371/journal.pbio.3001010 (PMC7793244; doi:10.1371/journal.pbio.3001010)
Supplement: S3 Text — (PDF) [file pbio.3001010.s003.pdf]

Supplemental Material to "Long-term experimental evolution of HIV-1 reveals effects of environment and mutational history"

3 Analysis of growth rates in the HIV-LTE

Measurements of growth rates were performed for the ancestor NL4-3 and 4 timepoints of 4 of the evolutionary lines. Generally, growth rates increased in later timepoints (as indicated by a significant increase in the area under the growth curve over time, see figure B), and adaptation went beyond the own environment.

Figure Aa makes it seem like the virus initially grows much better than MT-2. However, this comparison cannot be made since the cells might be in different conditions and this difference was not observed in similar experiments done earlier (see for example Figure **??**).

In some of the lines, virus concentration drops at day 4. We suspect at this point, there are no cells left to infect due to the earlier virus production in these lines. This indicates that in some lines, selection seems to be directed towards earlier virus production rather than more virus production.

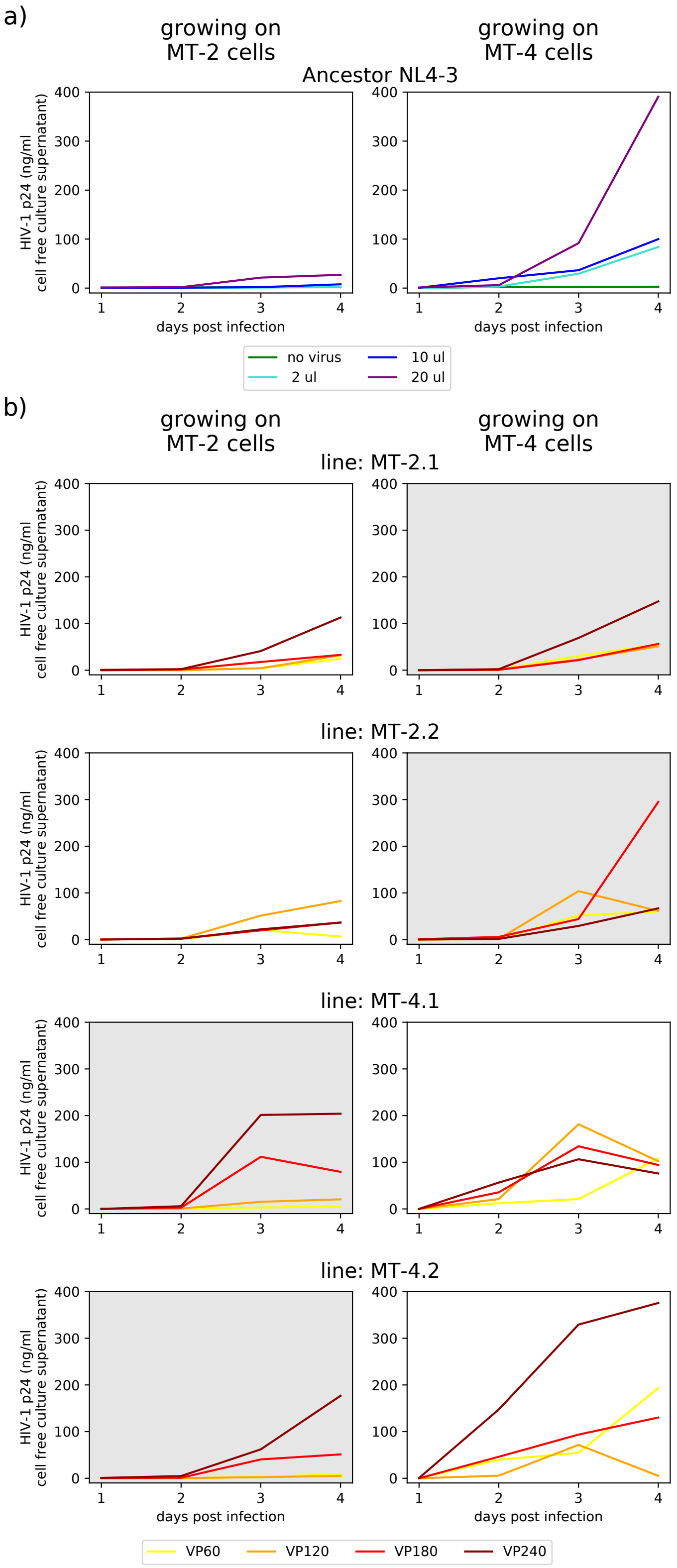

Figure A: Replication kinetics of the virus growing on MT-2 cells (left) or MT-4 cells (right). a) replication kinetics of different inoculum sizes for the ancestor NL4-3. b) replication kinetics of passages 60,120, 180 and 240 of evolved lines MT-2.1, MT-2.2, MT-4.1 and MT-4.2. Shaded backgrounds indicate growth on cells different from the ones the line evolved on.

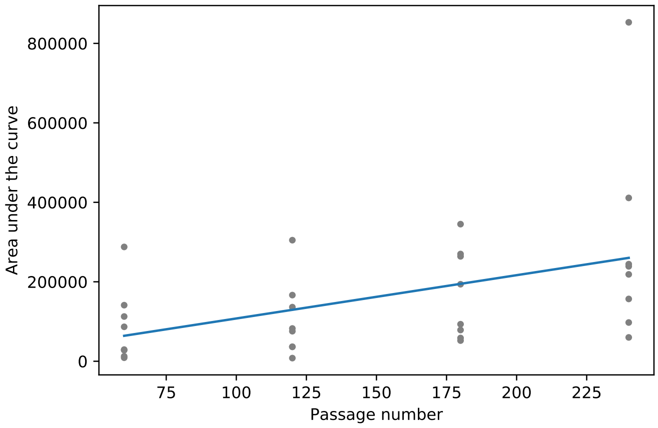

Figure B: Area under the curve for each of the growth rate assays in Figure A vs. the passage number. Linear Regression (blue line) results in a significantly positive slope (p-value: 0.01), indicating that the growth rate increases with time.
