# Supplementary material for: Long-term experimental evolution of HIV-1 reveals effects of environment and mutational history
Source: PLoS Biol. 2020 Dec 28;18(12):e3001010. doi: 10.1371/journal.pbio.3001010 (PMC7793244; doi:10.1371/journal.pbio.3001010)
Supplement: Attachment — Submitted filename: Response to reviewers.pdf [file pbio.3001010.s004.pdf]

We would like to thank all the reviewers for their insightful comments.
We have rewritten several sections in order to be more complete and to provide a better overview of how this study fits in the existing literature on viral evolution and other experimental evolution studies.
Below, we address the specific points raised by the academic editor and the reviewers comments point by point.

Points from the academic editor:
a) Please could you make quantitative predictions of the number of fixed and segregating alleles in your populations, assuming a neutral (mutation/drift) model, and compare these predictions against the observed numbers of mutations? (this is an extension of a request made on a previous version of the manuscript that was not reviewed)

> **In section 2.1, we already describe the number of expected majority mutations and shared mutations in a neutral model, when using two different mutation rates. We have now completed this with the expectation for fixed mutations (reaching a frequency of more than 0.99).**
> **While the number of majority mutations in the data and in a neutral model with mutation rate 10^{-5} is very similar, the number of fixed and shared mutation is quite different. Even when the mutation rate is 10x higher, we don't see as many fixed or shared mutations. While the higher number of fixed mutations could be due to unexpectedly small bottlenecks, we believe the high number of shared mutations indicates selection.**

b) Rather than performing competition assays, as suggested by reviewer #3, rewording should suffice (e.g. "growth rate" instead of "fitness").

> **We are now more careful with the wording throughout the manuscript.**

Reviewer's comments:
Reviewer #1:
In this second version of the manuscript the authors address the concerns raised by the journal editors, first, whether drift or selection was the main driver of evolution is not yet convincing, with detailed selection analyses and direct fitness assays needed, second, the reasons why diversity and evolutionary rate were cell type-dependent remain obscure.

The main conclusion of this study is that the HIV-1 fitness landscape is mostly smooth, with many paths towards the same fitness peak, that is, the same adaptive mutations are fixed in independent evolutionary lines. This finding is not surprising since virus propagation in cell culture is not neutral, viruses are subjected to the selective forces exerted by the cell. As showed HIV-1 drug selection there is clear convergent evolution in selecting resistance, similarly, virus coreceptor switch from an R5 to X4 tropism that occurs in most infected patients through infection and disease progression is also an experiment of convergent phenotypic and genetic evolution, moreover, resistance selection and coreceptor switch are observed in the same time scale in most infected patients.

Regarding the reasons why diversity and evolutionary rate were cell type-dependent is not also surprising since evolutionary forces may be different between cell types and the virus may also respond differently, however, these two cell types also share similar evolutionary forces (e.g. innate response, restriction factors, etc) and therefore, as showed here, at the end of the experiment viruses passaged in different cell types share most of the observed mutations. Say that, although with some concerns (see below), this reviewer believes that this is an interesting paper that may seed further evolutionary studies and, therefore, merits its publication on Plos Biology.

We thank the reviewer for these excellent points. We fully agree that drug selection, co-receptor switch, etc. are known to constitute selective pressures leading to parallel evolution. We would like to emphasize, however, that our experiments were designed to be as close to neutral as possible. The used viral strain (HIV-1 NL4-3) is known to replicate very well in the two cell lines used.

There is no drug pressure, no adaptive immune response and no possibility of target cell depletion. We find it surprising to see this amount of parallel evolution, indicating continued adaptation over years of growth in the same environment.

Specific comments

1. As a virologist it is unclear for me whether the virus fitness differences displayed in figure S5 are statistically significant. The authors should calculate the slopes of the virus kinetic experiments and confront them in a statistical test. To this end, replicas of the kinetic experiments should be performed.

By aggregating the different kinetic experiments, we can assess whether growth rate increases over time. For this, we have calculated the area under the curve for each of the growth rate assays. Linear regression on these data points indicates that growth rates increase significantly (p-value of the slope: 0.01) over time. We have added a figure in the supplemental material to illustrate this point.

We are currently planning dual infection/competition experiments to determine viral fitness in more detail and accuracy. We planned for this to be a separate analysis and to present these results in a second manuscript.

2. Why are synonymous mutations shared in different experiments? (last paragraph on page 5) Are they adaptive? The authors may discuss this finding, are they affecting virus fitness?

The highly shared nature of some of these 'silent' mutations -- synonymous mutations are shared in up to 10 lines -- indicates that they are likely adaptive, as these high levels of sharing are unlikely to happen under neutral conditions (see section 2.1). Synonymous mutations being under selective pressure in HIV is not a new finding (see for example Zanini & Neher, J Virol, 2013), and could for example be due to RNA structure and codon optimization.

We have added a paragraph to the discussion exploring the potential cause of the highly shared mutations that are synonymous or in non-coding regions.

3. MT4 and MT2 cells express ZAP, is pNL4-3 getting rid of CpG pairs through passages? Authors may discuss on virus nucleotide and dinucleotide frequencies.

The HIV genome contains very little CpG sites to begin with (Alinejad-Rokny et al. MBE, 2016) and we see no significant change in CpG frequency at the final time point compared to the ancestor. In fact, while almost all other dinucleotide frequencies change in line with the nucleotide frequencies (which change due to somewhat uneven mutation rates), CpG remains remarkably constant, indicating that those remaining CpG sites are likely conserved sites.
We have included these results into the supplementary material.

4. Finally, I am guessing whether it would be of interest to employ not only two different cell types but also two different virus genetic backgrounds.

We agree that it would be very interesting to additionally study different genetic backgrounds for the virus. Unfortunately, this is well outside the scope of this paper, and would require another 2-3 years of experiments.

5. There are some in silico methods, simulations of neutral sequence evolution and randomization tests, that I can not evaluate because I am not familiar with them.

Reviewer #2:
Major comments:
1. I have some concerns about the extent of new ground broken by the study. The main findings (high degree of parallelism in evolved virus lines, virus evolution shaped by environment and historical contingency) are similar to observations from previous experimental evolution studies with RNA viruses (e.g. Sanjuan et al. 2004; Sanjuan et al. 2005; da Silva et al. 2010; Morley and Turner 2017). The long-term nature of this experiment does make it very unique, but I felt an opportunity was missed to foreground what this long-term experiment can teach us relative to typical shorter evolution experiments (see Wiser et al. 2013; Tenaillon et al. 2016 for examples of these long-term patterns from Lenski's LTEE).

> **We agree that many of our results are consistent with previous findings from smaller passaging experiments and point-directed mutagenesis studies, like the ones pointed out by the reviewer. However, there are several unique aspects about our long-term setup that allow for novel insights. The experiment was set up with minimal selection pressures (the viral ancestor replicates well in both cell types). Interestingly, we see no saturation of mutations: even after several years of adaptation, new - likely beneficial - mutations keep appearing. We think this is an important insight into the adaptability of HIV. Additionally, the large amount of mutations that occur across the different experimental lines allow for more rigorous statistics. The analyses presented in this paper with respect to untangling the effects of history and environment, would not be possible in a smaller-scale experiment, because the statistical power of many mutations is needed.**
> **We have extended our discussion to highlight the benefits of the long-term nature of this study.**

2. This article included descriptions of temporal patterns of molecular evolution (section 2.3), but was lacking in quantitative and statistical comparisons. For example, there was no statistical comparison of the curves in Figure 2 to support the observations of differences between treatments. The dynamics of selective sweeps in Figure 3 could also be described quantitatively (see Lang et al. 2013; Levy et al. 2015; Acevedo et al 2014; McDonald et al. 2016).

> **Statistically comparing the full time series in Figure 2 is difficult due to the non-linear nature of the mutations accumulation curves. However, a two-way ANOVA showed significant contribution of both the original and final environment of the final (passage 240) mutation counts, for both minority and majority mutations. We have included these results into the manuscript.**
> **For Figure 3, we deliberately did not perform a quantitative analysis similar to those described in the papers mentioned. In our data set, we cannot differentiate between ‘real’ mutation increases and linkage or clonal interference. The cited papers either employ an organism with lower mutational supply (and more sweep-like dynamics, where linkage and clonal interference can be neglected), or advanced sequencing techniques to catch very low-frequency mutations.**

4. The main question in this study is how environment and historical contingency interact to shape evolutionary trajectories. Differences in evolutionary history are built into the experiment by incorporating derived virus lines that are switched into different environments. However, with the exception of Figure 2, lines with different histories were not separated out during analysis. This makes the effect of evolutionary history less clear.

We fully agree that it would be preferable to separate out the different histories in our analyses. Unfortunately, the environmental switch happened - in retrospect - too early, so that not many mutations were fixed. This means that the signal from the switch is too weak to use in any of our analyses.

Minor comments:

1. In the first paragraph of the introduction, the authors state "Due to its high mutation rate, small genome, and large population sizes, it can quickly adapt to these changing selection pressures." It is not clear to me that a small genome results in quicker adaptation.

The effect of small genomes on adaptation is rather indirect. Genome size and mutation rate go hand in hand: a smaller genome allows for higher mutation rates, while still avoiding error catastrophe. Additionally, smaller genomes typically have less redundancy, making the average effect of a mutation larger, thereby increasing the rate of adaptation.
To reduce confusion, we have deleted 'small genome sizes' from this sentence.

2. "A high amount of environmentally robust mutations will lead to high levels of parallel or convergent evolution…" Clarification is needed here. Is the 'high amount of robust mutations' referring to the fraction of all possible mutations that are environmentally robust?

When there are more environmental robust mutations (as a fraction), more mutations will have the same effect across environments. This will lead to a higher chance for parallel (or convergent) evolution. We have rephrased this sentence in the manuscript.

3. It would be helpful if figures were numbered in the order in which they are cited in the text. For example, Fig 6 is the second figure cited in the text.

We have changed the referencing of figures in the text. The numbering now corresponds to the order of citation.

4. It was unclear to me why fitness measurements were only reported for a subset of the experimental lines. For example, it would be interesting to see fitness data for the virus lines that experienced an environmental switch.

We initially decided not to do any additional experiments with lines MT-2.A, MT-2.B, MT-4.A, MT-4.B and their derived lines due to the observed cross-contaminations, deciding to focus on the more reliably independent lines instead.
Additionally, we are currently planning dual infection/competition experiments to determine viral fitness in more detail and accuracy, which we intend to be a separate analysis to be presented in a second manuscript.

5. In Section 2.2, the definition of 'reversions' requires clarification. Reversion mutations are defined in the text as 'mutations back to the consensus at positions where the ancestral virus differs from the consensus HIV sequence.' Do the authors mean mutations back to the ancestral sequence?

In this case, we mean mutations from the ancestral sequence to the overall HIV consensus ('the consensus of consensus'), at places where the ancestral sequence differs from the consensus of consensus. We have now clarified this in the manuscript.

6. The plateau in the left panel of Figure 2 is very interesting. What do the authors think is going on there? This is an intriguing pattern that merits further discussion.

We believe this plateau is due to selective sweep dynamics, which we already mention in the discussion (‘The later saturation of these minority mutations in MT-2 can be explained by the observed sweep dynamics in this line: the sweeps wipe out the diversity built-up faster than mutation can replenish them.’)
We now refer to this hypothesis more prominently in the results as well.

7. In the legend for Figure 2, please clarify whether mutations included in the accumulation curves are relative to the ancestral sequence or consensus sequence at a given time point.

These are relative to ancestral sequence. We have added this to the legend.

Reviewer #3:
The main claims of this paper, in brief, are that HIV long-term adaptation in different cell environments appears to be non-neutral, environment specific, and that a considerable degree of parallel evolution occurs in small environmental changes. Ultimately, the claims are not novel because each of these points have been made in many other virus/host environment systems; where this work differs is that the passaging series is much longer than most studies, spanning hundreds of passages.

For a broader audience, this paper is almost exclusively focused on HIV, but seems to want to address broad topics of virus evolution (environment-specific adaptation, role of genetic background in accumulation of mutations, parallel evolution). A few references are made, but there is a very large body of work that covers this in many other viruses (and even other microbes). The authors could rewrite some parts to place their work in this broader context and make the paper more interesting to others.

> **In this study, we indeed try to assess general questions of virus evolution in the context of HIV. Since there is - as the reviewer mentions - a huge body of work on the topics discussed in our paper, we did not want to turn the introduction or discussion into a review paper. We do agree that the discussion was too concise, and have added several paragraphs discussing how our work fits into the broader world of experimental evolution in viruses and contrast it to the evolutionary dynamics of bacteria and yeast.**

Some other points to consider:
The two host environments are very similar environments. They are the same type of cell from two different origins. Most other work in virus evolution in different host environments uses different cell types, or even different host organisms - the difference between two clones of one cell type would not be considered 'significantly' different by many virologists, so I suggest providing information on how these two environments really differ from each other if it is known, or to include a comment on how little they differ in the discussion. Parallel evolution in this case would be expected.

> **It is correct that the two environments constitute similar cells from different donors. We do not know much about their expression profiles beyond what we mention in the manuscript with respect to differences in APOBEC3G expression. It is thus conceivable that they also differ in regard to many other aspects. In vivo, HIV-1 also infects a very limited number of cell types, mainly CD4+ T-cells and macrophages. The differences in environments here are similar to the differences in the natural environment of HIV-1.**
>
> **While we are not exactly sure of the exact differences in selection pressures the virus experiences, we do not expect them to be very large, since the virus already replicates well in both cell lines. Over the course of the experiment, selection pressures were kept low by design. We think this constitutes a major finding of this experiment: selection pressures, and selection differences, do not need to be very large to have a measurable effect on the evolutionary genetics of a virus.**
>
> **We have now expanded on the differences and similarities between the environments in the discussion.**

The fitness measures of fig S5 are not relative fitness measures, no competition experiments are performed, they are only replication kinetic experiments. It is known that viruses with small, yet significant, fitness differences have the same replication kinetics when they are grown alone; but a direct competition assay would reveal these differences. This test could be possible with a genetically marked NL4.3 clone. I leave it up to the authors and editor to determine whether this is required. In absence, I would think it safer to refer to the work as measures of replication, and not measures of fitness.

> **We agree that this is Indeed rather a measure of growth rates than of true relative fitness. We are now more careful with nomenclature throughout the manuscript.**

Finally, it is important to emphasize that the study uses only a single, lab adapted strain (NL4.3). All of the observations and conclusions should be viewed with this in mind, and this should be clearly addressed in the discussion. NL4.3 is not a representative of most HIV strains carried by individuals, and is already well adapted to growth in cell culture conditions.

> **The use of a well-adapted strain was by design, as we wanted to observe evolution in HIV with minimal selective pressures. This way, we are able to answer more fundamental questions about evolution in HIV and viruses in general. The experimental design is, as highlighted in the discussion, far removed from real HIV infections, and we therefore do not see a problem using a strain that is not entirely representative of those strains carried by individuals, especially considering that - to our knowledge - HIV-1 NL4-3 did not show any uncommon phenotype not observed in other HIV-1 strains/clones.**
> **We are now highlighting the limitations of NL4-3 in the discussion.**

Two points I'd like to mention that I found quite interesting:
-The authors show that APOBEC-induced mutations are not relevant among mutations that reach majority.
-In section 2.3, the data nicely show that when one passage series is switched to the other cell type, the appearance of >5% minority mutations is specific to cell type. These results are very clear and really shows how even a small difference in environment can have a primary influence on a virus's evolution.
