# Supplementary material for: Long-term experimental evolution of HIV-1 reveals effects of environment and mutational history
Source: PLoS Biol. 2020 Dec 28;18(12):e3001010. doi: 10.1371/journal.pbio.3001010 (PMC7793244; doi:10.1371/journal.pbio.3001010)
Supplement: Attachment — Submitted filename: reviewer_response.pdf [file pbio.3001010.s005.pdf]

No responses to reviewers are needed in this version.
